# Supplementary material for: Sexual-risk behaviours and HIV and syphilis prevalence among in- and out-of-school adolescent girls and young women in Uganda: A cross-sectional study
Source: PLoS One. 2021 Sep 10;16(9):e0257321. doi: 10.1371/journal.pone.0257321 (PMC8432796; doi:10.1371/journal.pone.0257321)
Supplement: S1 Questionnaire — (PDF) [file pone.0257321.s002.pdf]

FORMATIVE ASSESSMENT OF ADOLESCENT GIRLS AND YOUNG WOMEN'S HIV, GENDER-BASED VIOLENCE AND SEXUAL AND REPRODUCTIVE HEALTH STATUS

QUESTIONNAIRE FOR 10-24 YEAR-OLD ADOLESCENTS AND YOUNG WOMEN

IDENTIFICATION OF THE PARTICIPANT	
DISTRICT CODE	☐☐
INTERVIEWER CODE	☐☐☐
NAME OF VILLAGE: ____________________ (For out-of-school respondents)	
NAME OF SCHOOL: ____________________ (For in-school respondents)	
PARTICIPANT NUMBER e.g. AGYW01001 (01=district code, 001=participant's number)	AGYW ☐☐☐☐☐
DATE	☐☐☐ dd/mm/yyyy
SCHOOLING STATUS: 1…… IN-SCHOOL 2 ……. OUT OF SCHOOL	☐
<u>FOR SCHOOL-BASED INTERVIEWS:</u><br>SCHOOL OWNERSHIP: 1… PUBLIC 2…FAITH-BASED/NGO 3…. PRIVATE<br>SCHOOL LOCATION: 1 …RURAL 2…… TOWNSHIP 3 …. OTHER<br>(SPECIFY) ____________________	☐<br>☐
SCHOOL LEVEL (FOR SCHOOL-BASED INTERVIEWS ONLY):<br>PRIMARY SCHOOL ……………………………………………1<br>SECONDARY SCHOOL...2<br>VOCATIONAL/TERTIARY INSTITUTION ………………………………….... 3<br>NOT APPLICABLE (NEVER WENT TO SCHOOL)………………………4	☐

INTRODUCTION

Ms _____________________ (DO NOT WRITE THE NAME, JUST MENTION)

As mentioned in the form you have signed, I am called ________________ and am here on behalf of Makerere University School of Public Health and The AIDS Support Organization (TASO). I am part of a team that is collecting data on HIV and sexual, gender based violence (GBV) and sexual reproductive health (SRH) status among adolescent girls and young women (AGYW) in this district. We very much appreciate your participation in this survey. This information will help the government to plan HIV, GBV and SRH services for AGYW better. The interview will take around 1 hour. Whatever information you provide will be kept strictly confidential and will not be shown to any other unauthorized person.

May I begin the interview now?

Respondent agrees to be interviewed…….1 respondent does not agree to be interviewed…….2

INTERVIEW STARTED AT: ______AM/PM

100. GENERAL CHARACTERISTICS OF THE RESPONDENTS

No.	QUESTION	CODING CATEGORIES	SKIP
101.	In what month and year were you born?	MONTH …………………………<br>DON'T KNOW MONTH…….98<br>YEAR ……………………………<br>DON'T KNOW YEAR……9998	
102.	How old were you at your last birthday? *VERIFY AGE WITH MONTH AND YEAR OF BIRTH IN Q101.*	AGE IN COMPLETED YEARS<br>DON'T KNOW…………….98	
103a	Have you ever attended school?	YES……………………………………………………………….1<br>NO……………………………………………………………….2	**If 2 GOTO Q106**
103b	Are you still in school? (***Being in school here refers to attending formal classes (e.g. P1 or S1)excluding vocational studies)***	YES……………………………………………………………….1<br>NO……………………………………………………………….2	**If 1, GOTO Q105**
104a	What is the highest level of school you attained: *Primary, `O` level, `A level, university or other tertiary?*	PRIMARY (P.1 –P.7)…………………………………………….2<br>PRIMARY PROFESSIONAL …………………………………..3<br>O' LEVEL (S.1 – S.4) ……………………………………….4<br>O' LEVEL PROFESSIONAL ……………………………….5<br>A' LEVEL (S.5 – S.6) …………………………………….6<br>UNIVERSITY…………………………………………………7<br>OTHER TERTIARY (AFTER S.6) ………………………….8<br>OTHER (Specify) ________________________________9	
104b	How long has it been since you left school?	LESS THAN A YEAR ………………………………………1<br>1 YEAR AGO……………………………………………….2<br>2 YEARS AGO……………………………………………..3<br>3 YEARS AGO……………………………………………..4<br>4+ MORE YEARS AGO…………………………………….5<br>DON'T KNOW……………………………………………..6	
104c	What are some of the main reasons you are not in school? (**Select all that apply**)	Yes No<br>LACK OF SCHOOL FEES, UNIFORM OR MATERIALS …….1 2<br>GOT PREGNANT………………………………………….1 2<br>GOT MARRIED…………………………………………..1 2<br>GOT SICK……………………………………………….1 2<br>NEEDED OR WANTED TO EARN MONEY………………1 2<br>NOT A GOOD STUDENT/FAILED IN SCHOOL…………1 2<br>NOT INTERESTED IN SCHOOL…………………………1 2<br>OTHER REASON ……………………………………….1 2<br>(Specify ______________________________________)	
104d	If there was an opportunity for you to return to school, would you be willing to do so?	YES ……………………………………………………………..1<br>NO ……………………………………………………………..2	**IF 2, GOTO Q106**
104e	If yes, would you like to return to formal or vocational education?	FORMAL EDUCATION …………………………………………1<br>VOCATIONAL EDUCATION …………………………………….2	**Skip to Q105a**

No.	QUESTION	CODING CATEGORIES	SKIP
105	If in school, in which class are you? (***Indicate actual class if below S6; otherwise, code 88 for University or 89 for other tertiary level***)	CLASS [ ][ ]<br>UNIVERSITY ………………………………………….……………. 88<br>OTHER TERTIARY ………………………………………………… 89	
105a	Are you able to read and write in English?	YES ……………………………………………………………………..1<br>NO ………………………………………………………………….….2	**If 2, GOTO Q105c**
105b	If yes, please read the following text: "*The consequences of illiteracy are many and harmful in several respects. As well as affecting illiterate individuals themselves in their daily lives and often jeopardizing their future, this scourge has a significant effect on society, both socially and economically* "<br>**Interviewer**: Has the participant been able to read the text?	YES, BUT WITH DIFFICULTY…………………………………….…..1<br>YES, WITH EASE (PROPER PRONOUNCIATION)………………….2<br>NO, SHE WAS NOT ABLE TO READ THE TEXT……………………3	**IF 3, ask Q105a again and code accordingly**<br><br>**Skip to Q106 if out-of-school**
105c	**Interviewer: Ask Q105c if respondent is in-school. Else, skip to Q106.**<br><br>During the past month, how many days did you miss school for any reason except for when school was closed or for holidays?	NONE …………………………………………………………….…..…1<br>1-2 DAYS………………………………………………….…………..2<br>3-5 DAYS ……………………………………………………….……….3<br>MORE THAN 5 DAYS …………………………………………………4<br>DON'T KNOW…………………………………………………………5	**IF 1, GOTO Q106**
105d	What were the main reasons you missed school last month? (**Circle all that apply**)	Yes No<br>I WAS SICK………………………………………………………..…1 2<br>LACK OF SCHOOL FEES …………………………………..…. 1 2<br>HAD MY PERIOD ……………………………………………....1 2<br>HELPING OUT AT HOME………………………………………1 2<br>BABYSITTING YOUNGER BROTHERS/SISTERS …………1 2<br>WORKING TO EARN MONEY…………………………………1 2<br>HANGING OUT WITH FRIENDS………………………………1 2<br>AT MY BOYFRIEND'S PLACE……………………………… 1 2<br>STUDYING FOR AN EXAM ……………………….……… 1 2<br>OTHER (SPECIFY) _______________________________1 2<br>DON'T KNOW 1 2	
106	Who do you live with?	ALONE ……………………….………………………….…………..1<br>FRIENDS………………………………………………………….2<br>MOTHER ALONE……………………………………………….3<br>FATHER ALONE………………………………………………...4<br>BOTH PARENTS………………………………………………...5<br>WITH OTHER RELATIVES…………………………………….6<br>HUSBAND/PARTNER …………………………………………7	
107a	Are you currently married? *(by marriage I mean religious, traditional, civil or consensual union)*	NEVER MARRIED…………….……………………......1<br>IN RELATIONSHIP BUT NOT MARRIED…..........2<br>MARRIED/UNION……………………………………3<br>DIVORCED/SEPARATED………………..……..........4<br>WIDOWED…………………...……………..….........5	**IF 1,4,5 GOTO Q108**<br><br>**IF 2, skip to Q107c**
107b	Does your spouse have more than one woman he considers a wife?	MONOGAMOUS…………………………….……1<br>POLYGAMOUS…………………………...…..2	
107c	How old is your partner? **INTERVIEWER NOTE:** ***If not married, ask age for most recent sexual partner***	AGE IN YEARS [ ][ ]<br>DON'T KNOW 98<br>Not Applicable 97	
108	What is your religion?	CATHOLIC……………………………………...…......………...1<br>ANGLICAN/PROTESTANT…………….……...........……………2<br>MOSLEM…………………………………………........………3<br>PENTECOSTAL /BORN AGAIN / EVANGELICAL…......................4<br>SEVENTH DAY ADVENTIST……………………………...............5<br>ORTHODOX…………………………………………………………6<br>OTHERS(specify)………………………………......…….........7<br>____________________________________	

No.	QUESTION	CODING CATEGORIES	SKIP
109	What is your main occupation, where you spend most of your time?	PEASANT FARMER……………………………………...…...1<br>SALARIED……………………………………………………2<br>BUSINESS/COMMERCIAL……………………………………3<br>CASUAL WORKER……………………………………………4<br>HOUSE WIFE…………………………………………………5<br>PUPIL/STUDENT/NO INCOME………………………………6<br>OTHER (SPECIFY)…………………………………………7<br>______________________________	**IF 5, 6 GOTO Q111**
110	What is your estimated average monthly expenditure?	LESS THAN 10,000/=……………………………………1<br>10,000-30,000/=……………………………………………..2<br>31,000-50,000/=……………………………………………..3<br>51,000-100,000………………………………………………4<br>101,000-500,000………………………………………………5<br>>500,000………………………………………………………6	

No.	QUESTION		Yes	No	DK
111	Do you or does any member of your household own the following? **Please consider only <u>functional</u> items.** ***Interviewer: Read out options and check all that applies. Start with "A functional [name of item]"***	A radio……………………………………………………	1	2	98
		A television set………………………………………………	1	2	98
		A bicycle……………………………………………………	1	2	98
		A motor cycle………………………………………………	1	2	98
		Your own/family home………………………………………	1	2	98
		a cell phone…………………………………………………	1	2	98
		a regular (land line) phone ………………………………	1	2	98
		a computer …………………………………………………	1	2	98
		An income generating business …………………………	1	2	98
		An indoor bathroom ………………………………………	1	2	98
		Running water either inside the house or inside the compound of your house ………………………………	1	2	98
		Electricity …………………………………………………	1	2	98
		Car …………………………………………………………	1	2	98
		Generator …………………………………………………	1	2	98
		Solar…………………………………………………………	1	2	98

200. SOURCES OF INFORMATION ON AND KNOWLEDGE OF REPRODUCTIVE HEALTH (CIRCLE THE APPROPRIATE CHOICE)

Question	Source	(1) Most Important	(2) Second most important	(3) Preferred
201 Young people learn about **puberty** - I mean the ways in which boys' and girls' bodies change during the teenage years - from many sources. They may learn from teachers at school, parents, brothers and sisters, from friends, from doctors or they may learn from books, films and magazines. What has been the most important source of information for you on this topic? And the second most important? CIRCLE MOST IMPORTANT IN COL 1 AND SECOND MOST IMPORTANT IN COL 2	01.School teacher	01	01	01
	02.Mother	02	02	02
	03.Father	03	03	03
	04.Brother	04	04	04
	05.Sister	05	05	05
	06.Other family members	06	06	06
	07.Friends	07	07	07
	08. Health workers	08	08	08
	09.Books/magazines	09	09	09
	10.Films/Videos/TV	10	10	10
	11. Religious institutions	11	11	11
202 From whom, or where, would you prefer to have received more information on this topic? CIRCLE ONE ANSWER IN COL. 3	12. Social media	12	12	12
	13. Radio	13	13	13
	14. Clubs/entertainment	14	14	14
	15. Other (Specify)	15	15	15
		________	________	________
	16. Never had access			

Question	Response	(1) Most Important	(2) Second most important	(3) Preferred
203 Now I would like to ask you a similar question about sources of information on the **sexual and reproductive systems of men and women** - I mean where eggs and sperm are made and how pregnancy occurs. What has been the most important source of information on this topic? And the second most important? CIRCLE IN COLS. 1 AND 2.	01.School teacher	01	01	01
	02.Mother	02	02	02
	03.Father	03	03	03
	04.Brother	04	04	04
	05.Sister	05	05	05
	06.Other family members	06	06	06
	07.Friends	07	07	07
	08. Health workers	08	08	08
	09.Books/magazines	09	09	09
	10.Films/Videos/TV	10	10	10
204 From whom or where, would you prefer to receive (or prefer to have received) more information on this topic? CIRCLE ONE ANSWER IN COL. 3	11. Religious institutions	11	11	11
	12. Social media	12	12	12
	13. Radio	13	13	13
	14. Clubs/entertainment	14	14	14
	15. Other (Specify)	15 ___________	15 ___________	15 ___________
	16. Never had access			
205 Now there is a third similar question about sources of information on relationships - I mean how boys should **interact** with girls and vice versa. What has been the most important source of information on this topic? And the second most important? CIRCLE IN COLS 1 AND 2	01.School teacher	01	01	01
	02.Mother	02	02	02
	03.Father	03	03	03
	04.Brother	04	04	04
	05.Sister	05	05	05
	06.Other family members	06	06	06
	07.Friends	07	07	07
	08. Health workers	08	08	08
	09.Books/magazines	09	09	09
	10.Films/Videos/TV	10	10	10
206 From whom, or where, would you prefer to receive more information on this topic? CIRCLE ONE ANSWER IN COL. 3	11. Religious institutions	11	11	11
	12. Social media	12	12	12
	13. Radio	13	13	13
	14. Clubs/entertainment	14	14	14
	15. Other (Specify)	15 ___________	15 ___________	15 ___________
	16. Never had access			

Question	Response	Code	Skip
207 Some schools have classes on puberty, on sexual and reproductive systems and on relationships between boys and girls. Did you ever attend school classes on any of these topics?	Yes	1	**IF 1 GOTO Q209**
	No	2	
	Not sure	3	
	Never been to school	4	
208 If No, Not sure or Never been to school, would you attend classes on puberty, sexual and reproductive systems and on relationships between boys and girls?	Yes	1	**AFTER ASKING GOTO Q210**
	No	2	
	Not sure	3	
209 Do you think that there should be (more) classes on these topics, fewer classes or was the number about right?	More	1	
	Less	2	
	About right	3	

210 Now I have some other questions on sex and reproduction. I will read you some statements. Please tell me whether you think the statement is true, or false, or whether you don't know.<br><br>A woman can get pregnant on the very first time that she has sexual intercourse.	True False Don't Know/ Not Sure<br><br>1 2 98	
211 A woman stops growing after she has had sexual intercourse for the first time.	1 2 98	
212 Masturbation causes serious damage to health.	1 2 98	
213 A woman is most likely to get pregnant if she has sexual intercourse half way between her periods.	1 2 98	
214. **Now I am going to ask you other questions about your health.**<br><br>Apart from HIV/AIDS, there are other diseases that men and women can catch by having sexual intercourse. These are known as **sexually transmitted diseases (STDs)**. Have you heard of any of these diseases?	YES……..……..1<br>NO…………….2	**IF 2, GOTO Q219**
215. What are the signs and symptoms of a sexually transmitted disease in a woman? Choose as many as needed. Please circle a number.	YES NO<br>DISCHARGE FROM VAGINA ……………………..1 2<br>PAIN DURING URINATION ………………………..1 2<br>GENITAL SWELLING………………………………..1 2<br>ULCERS/SORES IN GENITAL AREA …………....1 2<br>VAGINAL ITCHING…………………………………..1 2<br>OTHER, **SPECIFY**: ______________________ 1 2	
216. Have you ever had a sexually transmitted disease? Please circle a number	YES…………....1<br><br>NO…………….2	**IF 2, GOTO Q219**
217a. Did you seek treatment for the sexually transmitted disease? Please circle a number.	YES…………..1<br><br>NO…………….2	
217b. How soon did you seek treatment after realizing that you had any STD?	SAME DAY …………………….…………………………..1<br><br>1-2 DAYS ……………………………………………………..2<br><br>3-5 DAYS………………………………………………………3<br><br>1 WEEK………………………………………………………..4<br><br>MORE THAN 1 WEEK……………………………………….5	
218. From where did you seek treatment? Please circle a number.	SHOP……………………………………………………….……1<br>PHARMACY…………………………………………….....…..2<br>GOVT. HOSPITAL/HEALTH CENTRE/CLINIC……..……..3<br>PRIVATE DOCTOR/NURSE/CLINIC……….………………4<br>HERBAL/ TRADITIONAL PROVIDER …………………… ..5<br>OTHER, **SPECIFY**: 6	
219. On a typical day, how many hours in total do you spend using social media, chatting with friends online, playing computer games or using other interactive media?	NONE………………………………………………………….……1<br>ABOUT 1 HOUR OR LESS……………………………….……2<br>ABOUT 2 HOURS………………………………………………3<br>ABOUT 3 HOURS………………………………………………4<br>BETWEEN 4 AND 5 HOURS…………………………….……..5<br>MORE THAN FIVE HOURS……………………………………6	**IF 1, GOTO Q221a**

220. What forms of social media do you use to interact with your friends?	Yes No<br>FACEBOOK……………………………………………… ..1 2<br>WHATSAPP……………………………………………… 1 2<br>TWITTER…………………………………………………1 2<br>INSTAGRAM……………………………………………..1 2<br>OTHER (Specify)……………………………………………1 2	
221a. Do you own a mobile phone?	YES…………………………………………………………...1<br>NO……………………………………………………………2	**IF 2, GOTO Q222**
221b. If yes, what type of phone do you have?	ORDINARY PHONE………………………………………………1<br>SMART PHONE WITH TOUCH SCREEN……………………..2<br>OTHER TYPE (SPECIFY) ___________________________3	
222. Have you ever heard of a social media platform called **<u>SafePal</u>**?	YES ………………………………………………………………..1<br>NO ……………………………………………………………………2	**IF 2, GOTO Q301**
223. If yes, what have you heard about this App?	Yes No<br>It is an App for reporting sexual violence ……………….1 2<br>It can be downloaded from Google Play Store ………...1 2<br>Other (specify) _______________________________ 1 2	
224. Have you ever used SafePal App to report sexual violence?	YES…………………………………………………………………1<br>NO…………………………………………………………………..2	**IF 2, GO TO Q301**
225. If yes, to which organizations did you report the sexual violence case?	Action Aid Uganda………………………………………………1<br>Reproductive Health Uganda…………………………………..2<br>FIDA Uganda ……………………………………………………3<br>Naguru Teenage Center ………………………………………..4<br>Other (specify)___________________________________ .5	
226. Were you helped after reporting the case to the organization mentioned in Q225 above?	YES ………………………………………………………………..1<br>NO…………………………………………………………………..2	

300: SEXUALITY, PREGNANCY PERCEPTIONS AND GENDER NORMS

Now I am going to read to you a series of statements that you can agree or disagree with or indicate that you don't know. The statements are drawn from what people believe about a number of things regarding sexuality, marriage and pregnancy

	Statements	Agree	Don't know/not sure	Disagree	
301	I believe there is nothing wrong with unmarried boys and girls having sexual intercourse if they love each other.	1	2	3	
302	I think that sometimes a boy has to force a girl to have sex if he loves her.	1	2	3	
303	A boy will not respect a girl who agrees to have sex with him.	1	2	3	
304	Most girls who have sex before marriage regret it afterwards.	1	2	3	
305	Most boys who have sex before marriage regret it afterwards.	1	2	3	
306	A boy and a girl should have sex before they become engaged to see whether they are suited to each other.	1	2	3	
307	I believe that girls should remain virgins until they marry.	1	2	3	
308	I believe that boys should remain virgins until they marry.	1	2	3	
309	It is sometimes justifiable for a boy to hit his girlfriend.	1	2	3	
310	Most of my friends think that one-night stands are OK.	1	2	3	
311	It's all right for boys and girls to have sex with each other provided that they use methods to stop pregnancy.	1	2	3	
312	Most of my friends who have sex with someone use condoms regularly.	1	2	3	
313	I am confident that I can insist on condom use every time I have sex.	1	2	3	

314	I would never contemplate having an abortion myself or for my partner.	1	2	3	
315	It is mainly the woman's responsibility to ensure that contraception is used regularly.	1	2	3	
316	I think that you should be in love with someone before having sex with them.	1	2	3	
317	I feel that I know how to use a condom properly.	1	2	3	
318	Most of my friends would never contemplate having an abortion for themselves or their partner.	1	2	3	
319	Men need sex more frequently than do women	1	2	3	
320	Most of my friends believe that you should be in love before you have sex with someone.	1	2	3	
321	I would refuse to have sex with someone who is not prepared to use a condom.	1	2	3	
322	I believe that one night stands are OK	1	2	3	
323	A girl can get HIV the first time she has sexual intercourse	1	2	3	
324a	A girl can get pregnant after kissing	1	2	3	
324b	A girl can get pregnant after hugging	1	2	3	
325	A girl can swallow aspirin tablets to protect against pregnancy	1	2	3	
326	Using a condom can protect against pregnancy	1	2	3	
327	Using a condom can protect against HIV	1	2	3	
328	A girl can swallow a contraceptive pill before having sex that will protect against HIV	1	2	3	
329	A girl can use herbs to prevent a pregnancy	1	2	3	
330	How many of your friends have had sexual intercourse? Would you say many, some, a few, or none?	Many ……………………………………..1<br>Some ……………………………………..2<br>A few ……………………………………..3<br>None ……………………………………..4<br>Not sure ………………………………… 98			

400. SEXUAL BEHAVIOUR

Now I am going to ask you questions about sexuality and some questions will be about your own private life. Some of these questions need to be rather detailed and personal. Since this survey is confidential and your name is not included on this paper, no one else will know or connect your answers with you. We would appreciate your participation in answering these questions as openly as possible.

No.	QUESTION	CODING CATEGORIES	SKIP
401	Have you ever had any sexual intercourse in your life? (By this, I mean when a man or boy puts his penis in a woman or girl's vagina)	YES ……………………………………....1<br>NO ……………………………………....2	**IF 2, GOTO Q501**
402	If yes, how old were you when you had sexual intercourse for the very first time?	AGE IN YEARS ………………. [ ][ ]<br>DON'T KNOW…..…………... 98	
403	Which person did you have sex with for the first time?	Boyfriend ………………………………………1<br>Husband ………………………………………..2<br>Stranger…………………………………………3<br>Brother…………………………………………4<br>Teacher………………………………………....5<br>Uncle……………………………………………..6<br>Father……………………………………………7<br>Other relative (specify)_________________..8<br>Other (Specify) ______________________.....9	
404	How old was the partner that you had sex with for the	Same age as me ………………………………1	

	first time?	Younger than me ……………………………….2<br>1-2 years older than me………………………..3<br>3-4 years older than me………………………..4<br>5 or more years older than me………………..5<br>Don't know……………………………………..6	
405	The first time you had sexual intercourse with someone; would you say you were willing, somewhat willing or not willing at all? Willing means you gave permission or said it was OK or that you did it because you wanted to and not because someone forced you to do it	Very willing ……………………………………1<br>Somewhat willing………………………………2<br>Not willing at all…………………………………3<br>Don't know……………………………………….4	
406	The first time you had sexual intercourse with someone; did you or your partner do anything to prevent pregnancy?	YES ……………………………………………1<br>NO ……………………………………………..2<br>DO NOT REMEMBER ………………………3	**IF 2, 3 GO TO Q408**
407	The first time you had sex, what did you use to avoid a pregnancy? (**Select all that apply**)	Yes No<br>MALE CONDOM ……………………. 1 2<br>PILL ……………………………………1 2<br>INJECTION ……………………………1 2<br>FEMALE CONDOM…………………..1 2<br>WITHDRAWAL ………………………..1 2<br>EMERGENCY CONTRACEPTION …1 2<br>IUD/COIL ………………………………1 2<br>IMPLANT ………………………………1 2<br>OTHER (SPECIFY) _____________ 1 2	
408	The first time you had sexual intercourse, were you under the influence of alcohol or drugs?	YES …………………………………………… 1<br>NO……………………………………………….2<br>Don't know/Don't Remember	
409	When was the last time you had sexual intercourse?	Within 1 week…………………………………1<br>Within 1 month…………………………………..2<br>>1-12 months ago……………………………...3<br>>12 months ago………………………………...4	**IF 4 GOTO Q412**
410	The number of sexual partners people have may differ from person to person. Some people report having had one sex partner, some 2 or more partners.<br>In the **PAST 12 MONTHS**, how many different partners have you had sex with?	_____________Partners |____|____| DON'T KNOW 98<br>DECLINED TO ANSWER 99	
411	In the past 12 months, how often did you use condoms with all these partners?	Always……………………………….1<br>Sometimes …………………………...2<br>Rarely…………………………………3<br>Never ………………………………….4	
412	What was your relationship with the person you most recently had sex with?	Boyfriend …………………………………………1<br>Husband ……………………………………….…2<br>Stranger ……………………………………….…3<br>Brother ……………………………………….…4<br>Teacher ………………………………………....5<br>Uncle …………………………………………...6<br>Father …………………………………………..7<br>Other relative (specify)_________________ 8<br>Other (Specify) _______________________ 9	
413	How old was the partner that you had sex with for the last time?	Same age as me ………………………………. 1<br>Younger than me ………………………………. 2<br>1-2 years older than me ………………………..3<br>3-4 years older than me ………………………..4<br>5 or more years older than me ………………..5<br>Don't know ……………………………………. 98	
414	Thinking of **THE LAST TIME** you had intercourse with this partner, did you (or your partner) use a condom?	YES ………………………………………1<br>NO …………………………………………..2	**IF 2 GOTO Q420**
415	Thinking of all the times you had intercourse with this partner **IN THE LAST 12 MONTHS**, would you say you used a condom all the time, sometimes, or never?	Never………………………………………..1<br>Sometimes ……………………………………..2<br>Always ……………………………………...3<br>NA (Had no sex in last 12 months)….4<br>Don't know/don't remember ……………...98	
416a	Some people are worried about sexually transmitted infections. How concerned are you or were you about getting an STI from your partner?	Very concerned …………………………………1<br>Somewhat concerned ………………………….2<br>Not really concerned …………………………..3<br>Not at all concerned …………………………..4<br>Don't know …………………………………….98	

416b	Some people are worried about pregnancy. How concerned are you or were you about getting pregnant?	Very concerned ……………………………………1<br>Somewhat concerned ………………………….2<br>Not really concerned ……………………………3<br>Not at all concerned ……………………………4<br>Don't know ……………………………………..98	
417	Are you currently using a condom?	YES ……………………………………….1<br>NO ………………………………………..2	**IF 2 GOTO Q420**
418	If you are currently using a condom; what is the reason for using a condom?	Family planning……………………….1<br>HIV/other STI prevention………..…….2<br>Both …………………….…………….3	
419	Are you using a male or female condom?	Male condom………………….…..……..1<br>Female condom……….………..........2<br>Both……………………..………....3	**AFTER ASKING, GOTO Q421**
420	Now I would like to read to you a list of reasons why some people say they do not use condoms. Please tell me if any of these are reasons why you and your partner did not use a condom the last time you had sex. ***Mark all that apply***		
	Your partner is HIV positive?	YES ……………………………………….1<br>NO ………………………………………..2	
	Your partner would become suspicious of your HIV status if you asked him/her to use a condom?	YES ……………………………………….1<br>NO ………………………………………..2	
	Your partner refused?	YES ……………………………………….1<br>NO ………………………………………..2	
	You don't like using condoms?	YES ……………………………………….1<br>NO ………………………………………..2	
	Your partner doesn't like condoms?	YES ……………………………………….1<br>NO ………………………………………..2	
	You or your partner want/s to have children?	YES ……………………………………….1<br>NO ………………………………………..2	
	You did/do not have condoms?	YES ……………………………………….1<br>NO ………………………………………..2	
	You don't know where to get condoms?	YES ……………………………………….1<br>NO ………………………………………..2	
	You or your partner had problems with condoms such as a rash or burning?	YES ……………………………………….1<br>NO ………………………………………..2	
421	At times or routinely, some people do have sex in exchange for gifts, money, or services.<br>In the last 12 months, did you have sex where you received something in exchange including gifts, money or services from any sexual partner?	YES ……………………………………….1<br>NO ………………………………………..2<br>DON'T KNOW ………………………….98<br>REFUSED TO ANSWER ..…………….99	
422	At times or routinely, some people do have sex in exchange for gifts, money, or services.<br>In the last 12 months, did you have sex where you gave something in exchange including gifts, money or services from any sexual partner?	YES ……………………………………….1<br>NO ………………………………………..2<br>DON'T KNOW ………………………….98<br>REFUSED TO ANSWER ..…………….99	

500: CONDOM USE ATTITUDES

501	**SEE 401**<br>Respondent has experienced sexual intercourse ↓	Respondent has not experienced sexual intercourse →			**IF NO INTERCOURSE GOTO Q601**
502	Have you ever experienced a condom that split or broke during sexual intercourse?	YES . . . . 1<br>NO 2			
503	People have different opinions about condoms. I will read out some opinions. For each one, I want you to tell me whether you agree or disagree, or whether you don't know	Agree	Disagree	Don't know/not sure	
504	Condoms are an effective method of preventing pregnancy	1	2	3	
505	Condoms can be used more than once	1	2	3	

506	A girl can suggest to her boyfriend that he uses a condom	1	2	3	
507	A boy can suggest to his girlfriend that he uses a condom	1	2	3	
508	Condoms are an effective way of protecting against HIV/AIDS	1	2	3	
509	Condoms are suitable for casual relationships	1	2	3	
510	Condoms are suitable for steady, loving relationships	1	2	3	
511	It would be too embarrassing for someone like you to buy or obtain condoms	1	2	3	
512	If a girl suggested using condoms to her partner, it would mean that she didn't trust him	1	2	3	
513	Condoms reduce sexual pleasure	1	2	3	
514	Condoms can slip off the man and disappear inside the woman's body	1	2	3	
515	If unmarried couples want to have sexual intercourse before marriage, they should use condoms	1	2	3	
516	Condoms are an effective way of protecting against sexually transmitted diseases	1	2	3	

600 THIS SECTION SHOULD BE ADMINISTERED TO RESPONDENTS THAT HAVE NEVER HAD SEX. FOR THOSE WHO HAVE EVER HAD SEX, SKIP TO SECTION 700

People may have mixed reasons for not having sexual intercourse. I will read out some reasons. Please tell me for each reason whether it applies to you or not.	Applies	Not applies	Don't Know/ Not Sure	
601. I don't feel ready to have sex.	1	2	98	
602. I have not had the opportunity.	1	2	98	
603. I think that sex before marriage is wrong	1	2	98	
604. I am afraid of getting pregnant	1	2	98	
605. I am afraid of getting HIV/AIDS or another sexually transmitted infection.	1	2	98	

606 And now I have a question about your future plans about sexual intercourse. Which of these statement best describes your plans? READ OUT<br><br>(a) I plan to wait until marriage<br>(b) I plan to wait until I am engaged to be married<br>(c) I plan to wait until I find someone I love<br>(d) I plan to have sexual intercourse when an opportunity comes along	(a) Marriage ……………………………………1<br>(b) Engagement ………………………………2<br>(c) Love …………………………………………..3<br>(d) Opportunity…………………..……………...4	
607 Do you feel any pressure from others to have sexual intercourse? CIRCLE	YES ……………………………………………...,1<br>NO …………………………………………….2	**IF 2 GOTO Q701**
608 From whom do you feel pressure? PROBE CIRCLE ALL THAT APPLY	Friends ……………………………………..…1<br>Relatives ……………………..……………2<br>Work colleagues…………………………..…...3<br>Partner/special friend ……..……………..…....4<br>Teachers …………………………………….5<br>Other person…………………………..…......6<br>If other, specify……………………………7	

700. CONTRACEPTIVE KNOWLEDGE AND USE

READ TO RESPONDENT: I am going to read to you methods used to prevent pregnancy and I request you to tell me whether you have ever heard or used and stopped using any of them, and if so, I will request you to tell me the reason why you stopped using them IF THE ANSWER TO THE QUESTION ON EVER USED AND STOPPED USING A METHOD IS 'NO', WRITE "NA" UNDER REASONS WHY THEY STOPPED USING THE METHOD AND THEN ASK ABOUT THE NEXT METHOD.

	Method of contraception	HAVE YOU EVER HEARD?	HAVE YOU EVER USED?	ARE YOU CURRENTLY USING?	FOR THOSE CURRENTLY USING, ARE YOU SATISFIED WITH THE METHOD
**701**	Female sterilization.....A (tying women's tubes)	YES…….1<br>NO……..2	YES….....1<br>NO……..2<br>NA……..3	YES…….1<br>NO……..2<br>NA……..3	YES…….1<br>NO……..2<br>NA……..3
	Male sterilization…….B (tying men's tubes)	YES…….1<br>NO……..2	YES….....1<br>NO……..2<br>NA……..3	YES…….1<br>NO……..2<br>NA……..3	YES…….1<br>NO……..2<br>NA……..3
	Pill……………………………C	YES…….1<br>NO……..2	YES….....1<br>NO……..2<br>NA……..3	YES…….1<br>NO……..2<br>NA……..3	YES…….1<br>NO……..2<br>NA……..3
	IUD…………………….D (the coil)	YES…….1<br>NO……..2	YES….....1<br>NO……..2<br>NA……..3	YES…….1<br>NO……..2<br>NA……..3	YES…….1<br>NO……..2<br>NA……..3
	Injectables……………….E	YES…….1<br>NO……..2	YES….....1<br>NO……..2<br>NA……..3	YES…….1<br>NO……..2<br>NA……..3	YES…….1<br>NO……..2<br>NA……..3
	Implants…………F (put in forearm)	YES…….1<br>NO……..2	YES….....1<br>NO……..2<br>NA……..3	YES…….1<br>NO……..2<br>NA……..3	YES…….1<br>NO……..2<br>NA……..3
	Condoms……… G (for the man)	YES…….1<br>NO……..2	YES….....1<br>NO……..2<br>NA……..3	YES…….1<br>NO……..2<br>NA……..3	YES…….1<br>NO……..2<br>NA……..3
	Female condom….H (for women)	YES…….1<br>NO……..2	YES….....1<br>NO……..2<br>NA……..3	YES…….1<br>NO……..2<br>NA……..3	YES…….1<br>NO……..2<br>NA……..3
	Diaphragm……………….I	YES…….1<br>NO……..2	YES….....1<br>NO……..2<br>NA……..3	YES…….1<br>NO……..2<br>NA……..3	YES…….1<br>NO……..2<br>NA……..3
	Foam/jelly……………….J	YES…….1<br>NO……..2	YES….....1<br>NO……..2<br>NA……..3	YES…….1<br>NO……..2<br>NA……..3	YES…….1<br>NO……..2<br>NA……..3
	Lactational amenorrhoea method…………………..K	YES…….1<br>NO……..2	YES….....1<br>NO……..2<br>NA……..3	YES…….1<br>NO……..2<br>NA……..3	YES…….1<br>NO……..2<br>NA……..3
	Rhythm method………L (Safe period, moon beads)	YES…….1<br>NO……..2	YES….....1<br>NO……..2<br>NA……..3	YES…….1<br>NO……..2<br>NA……..3	YES…….1<br>NO……..2<br>NA……..3

	Method of contraception	HAVE YOU EVER HEARD?	HAVE YOU EVER USED?	ARE YOU CURRENTLY USING?	FOR THOSE CURRENTLY USING, ARE YOU SATISFIED WITH THE METHOD
	Emergency contraception…….M	YES…….1<br>NO……..2	YES…..…1<br>NO……..2<br>NA……..3	YES…….1<br>NO……..2<br>NA……..3	YES…….1<br>NO……..2<br>NA……..3
	Withdrawal……..……N	YES…….1<br>NO……..2	YES…..…1<br>NO……..2<br>NA……..3	YES…….1<br>NO……..2<br>NA……..3	YES…….1<br>NO……..2<br>NA……..3
	Others (specify). This includes all traditional methods you know of.<br>____________________	YES…….1<br>NO……..2	YES…..…1<br>NO……..2<br>NA……..3	YES…….1<br>NO……..2<br>NA……..3	YES…….1<br>NO……..2<br>NA……..3
**702**	Some women use more than one method to prevent pregnancy. Are you currently or have you ever used the condom and any other modern method? **Note: Only check yes, if the other method is a modern FP method)**	YES CURRENTLY USING……………..…………….1<br>YES EVER USED BUT NOT CURRENTLY USING …..2<br>NO…………………………………………………3			**IF 2 OR 3 GOTO Q704**
703	Where did you obtain the method you are currently using?	GOVT HOSPITAL/HEALTH CENTRE …………...1<br>FAMILY PLANNING CLINIC ……………….…..2<br>PUBLIC FACILITY OUTREACH …………………3<br>VILLAGE HEALTH TEAM MEMBER……..……….4<br>PRIVATE HOSPITAL/CLINIC……………………....5<br>PHARMACY/DRUG SHOP ………………………6<br>NGO COMMUNITY BASED DISTRIBUTOR ………7<br>DON'T KNOW/DON'T REMEMBER……………....8<br>OTHERS SPECIFY……………………………...9<br>____________________________________				

	Method of contraception	HAVE YOU EVER USED AND STOPPED USING METHOD?	WHAT ARE THE REASONS WHY YOU STOPPED USING METHOD (*CODE REASON FROM LIST BELOW TABLE*)
**704**	Female sterilization…A (tying women's tubes)	YES……..1<br>NO……..2	
	Male sterilization…….B (tying men's tubes)	YES……..1<br>NO……..2	
	Pill……………………………C	YES……..1<br>NO……..2	
	IUD…………………………D (the coil)	YES……..1<br>NO……..2	
	Injectables……………….E	YES……..1<br>NO……..2	
	Implants………………….F (put in forearm)	YES……..1<br>NO……..2	
	Condoms…………………G (for the man)	YES……..1<br>NO……..2	
	Female condom……….H (for women)	YES……..1<br>NO……..2	
	Diaphragm………………..I	YES……..1<br>NO……..2	
	Foam/jelly………………..J	YES……..1<br>NO……..2	
	Lactational amenorrhoea method…………………..K	YES……..1<br>NO……..2	
	Rhythm method………L (Safe period, moon beads)	YES……..1<br>NO……..2	
	Emergency ontraception….N	YES……..1<br>NO……..2	
	Withdrawal…………….M	YES……..1<br>NO……..2	

REASONS FOR STOPPING USING METHOD

Infrequent sex / husband away…............1	Subfecund / infecund ....…............................2
Not menstruated since last birth..............3	Menopausal / hysterectomy…………….…...…..4
Breastfeeding 5	Respondent opposed.....................…...............6
Husband / partner opposed......................7	Others opposed ....…..............8
Religious prohibition................................ 9	Fear of side effects ....…...............10
Health concerns...................................... 11	Lack of access / too far......................…..........12
Costs too much 13	Preferred method not available......................14
Inconvenient to use 15	Interferes with body's processes 16

	Method of contraception	HAVE YOU EVER HEARD?	HAVE YOU EVER USED?	ARE YOU CURRENTLY USING?	FOR THOSE CURRENTLY USING, ARE YOU SATISFIED WITH THE METHOD
	Trying to conceive/pregnant………………17		Method failed/conceived while on method….18		

What would be your preferred methods of preventing pregnancy? Are you able to access them?

	Method of contraception<br>(PLEASE PROBE) Do not read	PREFERRED METHOD	ARE YOU ABLE TO ACCESS THE METHOD?
705	Female sterilization….A (tying women's tubes)	YES……..1<br>NO……...2	YES……………...1<br>NO……………….2<br>NA………………3<br>DON'T KNOW ….98
	Male sterilization…….B (tying men's tubes)	YES……..1<br>NO……...2	YES……………...1<br>NO……………….2<br>NA………………3<br>DON'T KNOW ….98
	Pill……………………………C	YES……..1<br>NO……...2	YES……………...1<br>NO……………….2<br>NA………………3<br>DON'T KNOW ….98
	IUD…………………………D (the coil)	YES……..1<br>NO……...2	YES……………...1<br>NO……………….2<br>NA………………3<br>DON'T KNOW ….98
	Injectables……………….E	YES……..1<br>NO……...2	YES……………...1<br>NO……………….2<br>NA………………3<br>DON'T KNOW ….98
	Implants………………….F (put in forearm)	YES……..1<br>NO……...2	YES……………...1<br>NO……………….2<br>NA………………3<br>DON'T KNOW ….98
	Condoms…………………G (for the man)	YES……..1<br>NO……...2	YES……………...1<br>NO……………….2<br>NA………………3<br>DON'T KNOW ….98
	Female condom……….H (for women)	YES……..1<br>NO……...2	YES……………...1<br>NO……………….2<br>NA………………3<br>DON'T KNOW ….98
	Diaphragm……………….I	YES……..1<br>NO……...2	YES……………...1<br>NO……………….2<br>NA………………3<br>DON'T KNOW ….98
	Foam/jelly………………..J	YES……..1<br>NO……...2	YES……………...1<br>NO……………….2<br>NA………………3<br>DON'T KNOW ….98
	Lactational amenorrhoea method…………………..K	YES……..1<br>NO……...2	YES……………...1<br>NO……………….2<br>NA………………3<br>DON'T KNOW ….98
	Rhythm method………L (Safe period, beads)	YES……..1<br>NO……...2	YES……………...1<br>NO……………….2<br>NA………………3<br>DON'T KNOW ….98
	Emergency contraception…….M	YES……..1<br>NO……...2	YES……………...1<br>NO……………….2<br>NA………………3<br>DON'T KNOW ….98
	Withdrawal…………….N	YES……..1<br>NO……...2	YES……………...1<br>NO……………….2<br>NA………………3<br>DON'T KNOW ….98
	Others (specify). This includes all traditional methods you know of.<br>___________________________________	YES……..1<br>NO……...2	YES……………...1<br>NO……………….2<br>NA………………3

	Method of contraception (PLEASE PROBE) Do not read	PREFERRED METHOD	ARE YOU ABLE TO ACCESS THE METHOD?
			DON'T KNOW ….98

800. HIV/AIDS – MISCONCEPTIONS, HIV TESTING, COMPREHENSIVE KNOWLEDGE

801	Have you ever heard of an infection called HIV, the virus that causes AIDS?	YES……………………………1<br>NO ……………………………..2	**IF 2, GOTO Q833**
802	If a man/woman has HIV, does his/her partner always have HIV?	YES……………………………1<br>NO ……………………………..2<br>DON'T KNOW ……………….98	
803	Is it possible for a healthy-looking person to have HIV?	YES……………………………1<br>NO ……………………………..2<br>DON'T KNOW ……………….98	
804	If a mother is HIV-positive, can she transmit HIV to her baby? (Unprompted)	YES……………………………1<br>NO ……………………………..2<br>DON'T KNOW ……………….98	
805	Do you know of a place(s) where people can get tested for HIV?	YES……………………………1<br>NO ……………………………..2	
806	Have you ever been tested for HIV?	YES……………………………1<br>NO ……………………………..2	**IF 2 GOTO Q818**
807	How many times have you been tested for HIV?	Number ________	
808	When was your last HIV test?	Less than 3 months ago ………………….1<br>3-5 months ago ……………………………2<br>6-11 months ago …………………………..3<br>1-2 years ago ………………………………4<br>More than 2 years ago ……………………5<br>Can't remember ……………………………6	
809	Are you willing to tell me the last HIV test result you received?	YES…………………………………………..…1<br>NO ……………………………………………2	**IF 2 GOTO Q811**
810	If yes, what was the result of that HIV test?	POSITIVE………………………………1<br>NEGATIVE ……………………………..2<br>INDETERMINATE………………………3<br>DID NOT RECEIVE RESULTS …………4	**IF 2, 3, 4 GOTO Q815**
811	Have you disclosed your HIV status to your sexual partner?	YES…………………………………….………1<br>NO …………………………………………..2<br>NO PARTNER/NOT APPLICABLE……..……3	
812	If your HIV status is positive, have you registered in a clinic for HIV care?	Yes …………………………1<br>No …………………………..2	**IF 2, SKIP TO Q815**
813	Are you currently on antiretroviral therapy?	YES……………………………1<br>NO ……………………………..2	**IF 2 GOTO Q815**
814	How long ago did you start taking ARVs to manage your HIV?	_____________Months<br>______________Years	
815	Have you asked your current partner if he has ever tested for HIV?	YES…………………………………….…………1<br>NO ……………………………………….…….2<br>HAVE NO PARTNER/NOT APPLICABLE.....3	**GOTO Q 818**
816	Do you know whether your current partner has ever tested for HIV?	YES……………………………………………1<br>NO ……………………………………………..2	
817	Have you ever tested together with your current partner as a couple?	YES……………………………………………1<br>NO ……………………………………………..2	
818	Can people reduce their chance of getting the HIV virus by having just one uninfected sex partner who has no other sex partners?	YES . . . . .1<br>NO . . . . 2<br>DON'T KNOW 98	
819	Can people get the AIDS virus from mosquito bites?	YES . . . . .1<br>NO . . . . 2<br>DON'T KNOW 98	
820	Can people reduce their chance of getting the AIDS virus by using a condom every time they have sex?	YES . . . . .1<br>NO . . . . 2<br>DON'T KNOW 98	

821	Can people get the AIDS virus by sharing food with a person who has AIDS?	YES . . . . .1<br>NO . . . . 2<br>DON'T KNOW 98	
822	Can people get the AIDS virus because of witchcraft or other supernatural means?	YES . . . . .1<br>NO . . . . 2<br>DON'T KNOW 98	
824	Can the virus that causes AIDS be transmitted from a mother to her baby:<br>During<br>pregnancy?<br>During	YES NO DK<br>DURING PREG . . ….. 1 2 98<br>DURING DELIVERY…. . .1 2 98<br>BREASTFEEDING … . 1 2 98	
825	Can someone prevent her/himself from acquiring HIV/AIDS through abstinence?	YES . . . . .1<br>NO . . . . 2<br>DON'T KNOW 98	
826	Can male circumcision help to reduce risk of acquiring HIV/AIDS?	YES . . . . .1<br>NO . . . . 2<br>DON'T KNOW 98	
827	Are there any special drugs that a doctor or a nurse can give to a woman infected with the AIDS virus to reduce the risk of transmission to the baby?	YES . . . . .1<br>NO . . . . 2<br>DON'T KNOW 98	
828	Would you buy fresh vegetables from a shopkeeper or vendor if you knew that this person had the AIDS virus?	YES . . . . .1<br>NO . . . . 2<br>DON'T KNOW 98	
829	If a member of your family got infected with the AIDS virus, would you want it to remain a secret or not?	YES, REMAIN A SECRET………… 1<br>NO . . . . 2<br>DK/NOT SURE/DEPENDS ..……… 98	
830	If a member of your family became sick with AIDS, would you be willing to care for her or him in your own household?	YES . . . . 1<br>NO . . . . 2<br>DK/NOT SURE/DEPENDS ……….. 98	
831	In your opinion, if a female teacher has the AIDS virus but is not sick, should she be allowed to continue teaching in the school?	SHOULD BE ALLOW ……………. 1<br>SHOULD NOT BE ALLOWED …… 2<br>DK/NOT SURE/DEPENDS ……… 98	
832	Should children age 10-14 be taught about using a condom to avoid getting HIV/AIDS?	YES . . . . 1<br>NO . . . . 2<br>DK/NOT SURE/DEPENDS ………… 98	
833	Apart from AIDS, have you heard about other infections that can be transmitted through sexual contact?	YES . . . . 1<br>NO 2	**IF 2, GOTO Q901**
834	Now I would like to ask you some questions about your health in the **last 12 months**. During the last 12 months, have you had a disease which you got through sexual contact?	YES . . . . .1<br>NO . . . . 2<br>DON'T KNOW 98	**CROSS-CHECK WITH Q216**
835	Sometimes women experience a bad-smelling abnormal genital discharge. During the last 12 months, have you had a bad-smelling abnormal genital discharge?	YES . . . . .1<br>NO . . . . 2<br>DON'T KNOW 98	
836	Sometimes women have a genital sore or ulcer. During the last 12 months, have you had a genital sore or ulcer?	YES . . . . .1<br>NO . . . . 2<br>DON'T KNOW 98	
837	CHECK 834, 835, AND 836:<br>HAS HAD AN INFECTION (ANY 'YES') ↓<br>HAS NOT HAD AN INFECTION OR DOES NOT KNOW →		**IF NO INFECTION OR DK**<br>**GOTO Q840**

838	The last time you had (PROBLEM FROM 834/835/836), did you seek any kind of advice or treatment?	YES . . . . 1<br>NO 2	IF 2 GOTO Q840
839	Where did you go?<br><br>Any other place?<br><br>PROBE TO IDENTIFY EACH TYPE OF SOURCE.<br><br>IF UNABLE TO DETERMINE IF PUBLIC OR PRIVATE SECTOR, WRITE THE NAME OF THE PLACE.<br><br>(NAME OF PLACE(S)	**PUBLIC SECTOR**<br>GOVERNMENT HOSPITAL . . . . .1<br>GOVT. HEALTH CENTER . . . . .2<br>STAND-ALONE VCT CENTER . . . . .3<br>FAMILY PLANNING CLINIC . . . . . 4<br>OUT REACH . . . . 5<br>VILLAGE HEALTH TEAM .... 6<br>OTHER PUBLIC________________7<br>(SPECIFY)<br><br>**PRIVATE MEDICAL SECTOR**<br>PRIVATE HOSPITAL/CLINIC .... 8<br>STAND-ALONE VCT CENTER ...... 9<br>PHARMACY/DRUG SHOP . ... 10<br>PRIVATE DOCTOR/NURSE/<br>MIDWIFE .... 11<br>OUT REACH . . . . 12<br>TASO . . . . 13<br>AIDS INFORMATION CENTRE ..... 14<br>OTHER PRIVATE/NGO/MEDICAL<br>________________________ 15<br>(SPECIFY)<br>OTHER __________________ 16<br>(SPECIFY)	
840	If a wife knows her husband has a disease that she can get during sexual intercourse, is she justified in asking that they use a condom when they have sex?	YES . . . . .1<br>NO . . . . 2<br>DON'T KNOW 98	
841	Is a wife justified in refusing to have sex with her husband when she knows he has sex with other women?	YES . . . . .1<br>NO . . . . 2<br>DON'T KNOW 98	
842	CHECK 107A:<br><br>CURRENTLY MARRIED/ LIVING WITH A MAN ↓ NOT IN UNION →		IF NOT IN UNION, GOTO Q901
843	Can you say no to your (husband/partner) if you do not want to have sexual intercourse?	YES . . . . 1<br>NO . . . . 2<br>DEPENDS/NOT SURE 8	
844	Could you ask your (husband/partner) to use a condom if you wanted him to?	YES . . . . 1<br>NO . . . . 2<br>DEPENDS/NOT SURE ... 8	

900. MENSTRUAL HYGIENE

901	Have you started to have your periods?	YES . . . . 1<br>NO 2	If No, skip to 1001
902	How old were you when you first got your periods?	AGE AT FIRST PERIOD ___________________	
903	The last time you had your period, what did you use to manage it?	Did not use anything ……………………………….…1<br>Disposable sanitary napkins/pads …………………2<br>Tampons/menstrual cups …………………….……..3<br>Cloth/cloth pads ………………………………..………..4<br>Other (specify) ____________________ 5	
904	Check **Q103B**. If still in school, ask: In the past month, how many days of schooling did you miss because of your period?	NONE ……………………………………………….…1<br>1-2 DAYS ……………………………………….…….2<br>3-4 DAYS ……………………………………….…….3<br>5-6 DAYS ……………………………………….…….4<br>7 OR MORE DAYS …………………………………5	If not in school, continue to 1001

1000. PREGNANCY AND ANTENATAL CARE

1001	Have you ever been pregnant and gave birth to a baby?	YES . . . . 1<br>NO 2	**IF 2 GOTO Q1102**
1002	At what age did you become pregnant for the first time?	AGE AT FIRST PREG … _____________	
1003	How did the first pregnancy end?	Had a miscarriage ………………………………..1<br>Aborted ……………………………………………2<br>Live birth ……………………………………………3<br>Still birth ……………………………………………4<br>Still pregnant ………………………………………5	
1004	Did you have any antenatal visit or care during the pregnancy for the first birth?	YES . . . . 1<br>NO 2	
1005	How many pregnancies have you had during your life time?	NUMBER OF PREGNANCIES _______________	
1006	Did you have any antenatal visit or care during the pregnancy for the last birth?	YES ……………………………….……………1<br>NO ………………………………..……..…..2	**IF 2 GOTO Q1008**
1007	How many times did you visit the antenatal clinic during your last pregnancy?	None ……………………………………………………0<br>1 time ……………………………………………………1<br>2 times……………………………………………………2<br>3 times……………………………………………………3<br>4 times …………………………………………………..4<br>5 or more times ………………………………………..5	
1008	If you did not attend any antenatal clinic, what were the reasons for this?	Partner did not give me money for transport ……… 1<br>I never had money for transport ………………………2<br>Didn't know that I was pregnant ………………………3<br>Did not know where to go for antenatal care ……….4<br>I didn't know that I needed to go for antenatal care...5<br>I feared to go for antenatal care ………………………6<br>Other (specify)____________________________7	
1009	Where did you have your last delivery?	Government health facility ……………………………1<br>Private health facility …………………………………..2<br>At home …………………………………………………3<br>Traditional birth attendant …………………………….4<br>Other (specify_______________________)	
1010	During any of the antenatal visits for your last birth were you given any information about:<br>Babies getting the AIDS virus from their mother?<br>Things that you can do to prevent getting the AIDS virus?<br>Getting tested for the AIDS virus?<br>Family planning information?<br>Where to deliver from?	YES NO DK AIDS<br>FROM MOTHER 1 2 98<br>THINGS TO DO 1 2 98<br>TESTED FOR AIDS 1 2 98<br>FAMILY PLANNING 1 2 98<br>DELIVERY INFORMATION 1 2 98<br>BIRTH PREPAREDNESS 1 2 98	
1011	Have you ever had an abortion?	Yes……………………………………………………………1<br>No……………………………………………………….…2<br>Don't know…………………………………………….……3	**If NO, skip to Q1101**

1012	How many times have you had an abortion?	Once ……………………………………………………….....1<br>2 times…………………………………………………….2<br>3 times…………………………………………………….3<br>4+ times…………………………………………………..4<br>Don't know/can't tell………………………………………5	
1013	When did you have your most recent abortion? (**Interviewer**: If respondent has ever aborted once, this should still be taken as the most recent abortion)	This week ……………………………………….........1<br>1 week ago…………………………………………………2<br>1 month ago………………………………………………..3<br>2-4 months ago……………………………………………4<br>5+ months ago ……………………………………………5<br>Don't know/Don't remember ……………………………..6	
1014	Where did you have your most recent abortion?	Home………………………………………………………..1<br>Traditional Birth Attendant………………………………..2<br>Private doctor ……………………………………………...3<br>Private health facility ……………………………………...4<br>Government health facility ………………………………5<br>Other place (specify)__________________________ 6	
1015	Was your most recent abortion spontaneous or induced by you?	Spontaneous………………………………………………1<br>Induced………………………………………………….. 2<br>Don't know …………………………………………………..3	
1016	Referring to your most recent abortion: How did you manage the after-effects of abortion?	Did not do anything ………………………………………..1<br>Bought drugs from a pharmacy…………………………..2<br>Sought treatment from a traditional healer …………….3<br>Sought treatment from a medical professional ……….4<br>Other (specify)______________________________....5	

1100. MARRIAGE AND SEXUAL ACTIVITY

NO.	QUESTIONS AND FILTERS	CODING CATEGORIES	SKIP
1101	How old were you when you first got married?	AGE (completed years) _______________	
1102	Do you know of a place where a person can get condoms?	YES ……………………………………… 1<br>NO 2	**IF 2 GOTO Q1104**

1102	Where is that?<br><br>Any other place?<br><br>PROBE TO IDENTIFY EACH TYPE OF SOURCE.<br><br>IF UNABLE TO DETERMINE IF PUBLIC OR PRIVATE SECTOR, WRITE THE NAME OF THE PLACE. (NAME OF PLACE(S)	**PUBLIC SECTOR**<br>GOVERNMENT HOSPITAL .... 1<br>GOVT. HEALTH CENTER … … … … … . .. . . 2<br>FAMILY PLANNING CLINIC........................... 3<br>OUT REACH ....4<br>VILLAGE HEALTH TEAM ....5<br>OTHER PUBLIC SECTOR ....6<br>(SPECIFY) ___________________________<br><br>**PRIVATE MEDICAL SECTOR**<br>PRIVATE HOSPITAL/CLINIC . . . . . 7<br>PHARMACY . . . . 8<br>PRIVATE DOCTOR . . . . 9<br>OUT REACH . . . . 10<br>NGO COMMUNITY BASED DISTRIBUTOR.......................................11<br>OTHER PRIVATE MEDICAL SECTOR _______________________12<br>(SPECIFY)<br><br>**OTHER SOURCE**<br>SHOP . . . . 13<br>RELIGIOUS INSTITUTION........................14<br>FRIENDS/RELATIVES . . . . 15<br>STREET VENDOR.............................. 16<br>LODGE.. 17<br><br>OTHER ___________________________ 18<br>(SPECIFY)	
1103	If you wanted to, could you yourself get a condom?	YES . . . . 1<br>NO . . . . 2<br>DON'T KNOW/UNSURE 98	**IF 1**<br>**GOTO Q1104**
	If you can't get a condom yourself, what is the reason for this?	I fear to ask for a condom ....1<br>I cannot afford the cost of condoms ....2<br>I don't know where to find them ....3<br>Place is very far ....4<br>My partner does not like condoms ....5<br>My religion does not allow me to use condoms…6<br>Other (specify ____________________)…..7	
1104	Sometimes a woman can have a problem of constant leakage of urine or stool from her vagina during the day and night. This problem usually occurs after a difficult childbirth, but may also occur after a sexual assault or after pelvic surgery.<br><br>Have you ever experienced a constant leakage of urine or stool from your vagina during the day and night?	YES . . . . 1<br><br>NO . . . . 2<br><br>DON'T KNOW 98	**IF 2, 98**<br>**GOTO Q1107**
1105	Have you sought treatment for this condition?	YES . . . . 1<br>NO 2	**IF 1**<br>**GOTO Q1107**
1106	Why have you not sought treatment?	DO NOT KNOW CAN BE FIXED . . .. 1<br>DO NOT KNOW WHERE TO GO . . .. 2<br>TOO EXPENSIVE . . . . 3<br>TOO FAR . . . . 4<br>POOR QUALITY OF CARE . . . . 5<br>COULD NOT GET PERMISSION . . .. 6<br>EMBARRASSMENT . . . . 7<br><br>OTHER ___________________________ 8<br>(SPECIFY)	
1107	In some countries, there is a practice in which a girl may have parts of her genitals cut. Have you ever heard about this practice?	YES . . . . 1<br>NO 2	**IF 2**<br>**GOTO Q1201**
1108	Have you yourself ever been circumcised?	YES . . .. 1<br>NO 2	

1109	Do you think that female circumcision should be continued, or should it be stopped?	CONTINUED . . . . 1<br>STOPPED . . . . 2<br>DEPENDS . . . . 3<br>DON'T KNOW 8	

1200: INTIMATE PARTNER VIOLENCE

	CHECK 107A:<br>IF MARRIED OR IN A RELATIONSHIP ↓ IF NOT IN A RELATIONSHIP →				**IF NOT GOTO Q1301**
1201	First, I am going to ask you about some situations which happen to some women. Please tell me if these apply to your relationship with your (last) husband/partner?	YES	NO	DK	
	a) He (is/was) jealous or angry if you (talk/talked) to other men?	JEALOUS 1	2	98	
	b) He frequently (accuses/accused) you of being unfaithful?	ACCUSES 1	2	98	
	c) He (does/did) not permit you to meet your female friends?	NOT MEET FRIENDS....1	2	98	
	d) He (tries/tried) to limit your contact with your family?	NO FAMILY 1	2	98	
	e) He (insists/insisted) on knowing where you (are/were) at all times?	WHERE YOU ARE.......1	2	98	
	**Now I need to ask some more questions about your relationship with your (last) husband/partner.**<br>**A Did your (last) husband/partner ever:**	**B How often did this happen the last 12 months: often, only sometimes or not at all?**			
1202	A Did your (last) husband/partner ever do any of the following things to you:	B How often did this happen during the last 12 months: often, only sometimes, or not at all?			
		OFTEN	SOME-TIMES	NOT IN LAST 12 MONTHS	

	a) push you, shake you, or throw something at you?	YES ....1 → NO 2	1	2	3	
	b) slap you?	YES ....1 → NO 2	1	2	3	
	c) twist your arm or pull your hair?	YES ....1 → NO 2	1	2	3	
	d) punch you with his fist or with something that could hurt you?	YES ....1 → NO 2	1	2	3	
	e) kick you, drag you, or beat you up?	YES ....1 → NO 2	1	2	3	
	f) try to choke you or burn you on purpose?	YES ....1 → NO 2	1	2	3	
	g) threaten or attack you with a knife, gun, or other weapon?	YES ....1 → NO 2	1	2	3	
	h) physically force you to have sexual intercourse with him when you did not want to?	YES ....1 → NO 2	1	2	3	
	i) physically force you to perform any other sexual acts you did not want to?	YES ....1 → NO 2	1	2	3	
	j) force you with threats or in any other way to perform sexual acts you did not want to?	YES ....1 → NO 2	1	2	3	

	**CHECK 1202:** **AT LEAST ONE 'YES'** ↓	**NOT A SINGLE 'YES'** →	**IF NOT A SINGLE YES GOTO Q1301**
1203	Did the following ever happen as a result of what your (last) husband/partner did to you: a) You had cuts, bruises, or aches?	YES .... 1 NO 2	
	b) You had eye injuries, sprains, dislocations, or burns?	YES .... 1 NO 2	
	c) You had deep wounds, broken bones, broken teeth, or any other serious injury?	YES .... 1 NO 2	
1204	Have you ever hit, slapped, kicked, or done anything else to physically hurt your (last) (husband/partner) at times when he was not already beating or physically hurting you?	YES .... 1 NO 2	**IF 2 GOTO Q1206**
1205	In the last 12 months, how often have you done this to your (last) husband/partner: often, only sometimes, or not at all?	OFTEN .... 1 SOMETIMES .... 2 NOT AT ALL 3	
1206	Does (did) your (last) husband/partner drink alcohol?	YES .... 1 NO 2	**IF 2 GOTO Q1208**
1207	How often does (did) he get drunk: often, only sometimes, or never?	OFTEN .... 1 SOMETIMES .... 2 NOT AT ALL 3	
1208	Are (were) you afraid of your (last) husband/partner: most of the time, sometimes, or never?	MOST OF THE TIME AFRAID .... 1 SOMETIMES AFRAID .... 2 NEVER AFRAID 3	

1209	In the last 12 months, how often has this person physically hurt you: often, only sometimes, or not at all?	OFTEN . . . . 1<br>SOMETIMES . . . . 2<br>NOT AT ALL 3	
	**CHECK 1001:**<br>**EVER BEEN PREGNANT** ↓ **NEVER BEEN PREGNANT** →		**IF NEVER GOTO Q1301**
1210	Has anyone ever hit, slapped, kicked, or done anything else to hurt you physically while you were pregnant?	YES . . . . 1<br>NO 2	**IF 2 GOTO Q1301**
1211	Who has done any of these things to physically hurt you while you were pregnant?<br><br>Anyone else?<br><br>RECORD ALL MENTIONED.	CURRENT HUSBAND/PARTNER . . . . 1<br>MOTHER/STEP-MOTHER . . . . 2<br>FATHER/STEP-FATHER . . . . 3<br>BROTHER . . . . 4<br>DAUGHTER/SON . . . . 5<br>OTHER RELATIVE . . . . 6<br>FORMER HUSBAND/PARTNER . . . . 7<br>CURRENT BOYFRIEND . . . . 8<br>FORMER BOYFRIEND . . . . 9<br>MOTHER-IN-LAW . . . . 10<br>FATHER-IN-LAW . . . . 11<br>OTHER IN-LAW . . . . 12<br>TEACHER . . . . 13<br>EMPLOYER/SOMEONE AT WORK . . . . . . 14<br>POLICE/SOLDIER . . . .15<br>OTHER ______________________________ 16<br>(SPECIFY)	

1300: USE AND PERCEPTION OF HEALTH SERVICES

1301	Have you ever visited a health facility or doctor of any kind to receive services or information on contraception, pregnancy, abortion or sexually transmitted diseases?	YES . . . . 1<br>NO 2	**IF 2 GOTO Q1401**
1302	How many times have you sought services or information from a doctor or a nurse for these services in the last twelve months?	Number of times |_____|_____| Did not seek care in last 12 months 00	**IF 0 GOTO Q1401**
1303	Thinking about your last visit, did you go to a government clinic, health centre or hospital or a private doctor or clinic?	Government ……………………………1<br>Private ………………………………… 2<br>Other ………………………………… 3	
1304	When you last saw a doctor or a nurse, what was your reason for going to see him/her?	Contraception ………………………..1<br>STD ………………………………….. 2<br>Gynaecological exam …………………3<br>Pregnancy test …………………………4<br>Pregnancy termination ………………….5<br>MCH …………………………………… 6<br>Other …………………………………… 7	
1305	At the facility where you usually go, do you see any posters on contraception?	YES . . . . 1<br>NO 2	
1306	Have you ever been given brochures on contraception?	YES . . . . 1<br>NO 2	
1307	Did you ever attend a talk on contraception?	YES . . . . 1<br>NO 2	
1308	Have you ever asked/requested for contraceptive services during the consultation?	YES . . . . 1<br>NO 2	

1309	Does the doctor or nurse talk to you about:<br>(a) Contraception?<br>(b) Sexually transmitted diseases?<br>(c) Pregnancy?	YES NO<br>1 2<br>1 2<br>1 2	
1310	Did you feel comfortable enough to ask questions?	1 2	**IF 2 GOTO Q1401**
1311	Were the questions you asked during the consultation answered adequately?	1 2	
1312	Is there enough confidentiality?	1 2	

1400. Vulnerability Index Tool

The Vulnerability Index (VI) is intended for the selection of vulnerable AGYW who will be supported through the AGYW Program. The tool helps to determine a household's level of vulnerability (slight, moderate, and critical) based on individual and household level questions you will ask across all core program areas.

INSTRUCTIONS: Please use the following indicators to pre-select households where the VI tool will be administered. Pre-selection of vulnerable households requires the participation of community members and community workers. This ensures that the selection process is conducted in an efficient and transparent manner if critically and moderately vulnerable are to be identified**.**

HOUSEHOLD VULNERABILITY SECTION

1401	Does the household have **ANY** adult member who has been very sick for at least three months during the past 12 months?<br>***(By very sick, I mean that the household head or any adult member was too sick to work or do normal activities around the house for at least three of the past 12 months)***	YES . . . . 1<br>NO 2	
1402	Does the household have **ANY** severely disabled person?<br>***(Applies to both children and adult household members)***	YES . . . . 1<br>NO 2	
1403	Does the household have children not currently enrolled in school?<br>(***Children between the ages of 6-17 years***)	YES . . . . 1<br>NO 2	
1404	Does the household have or care for any orphans?	YES . . . . 1<br>NO 2	
	**CHECK 1401 - 1404**<br>**If "Yes"** for **at least ONE** ↓	**If "No"** for **ALL** →	IF NO GOTO Q1501
	**CPA 1: ECONOMIC STRENGTHENING**		
1405	Who is the household head?	Father ……………………….…1<br>Mother………………………….2<br>Relatives……………………….3<br>Children (6 – 17) years ……..4<br>Grand or Elderly Parents…….5<br>Partner/Spouse ………………6<br>Self ……………………………7	
1406	What is the highest education level of the household head?	PRIMARY (P.1 –P.7)…………….……..2<br>PRIMARY PROFESSIONAL ………....3<br>O' LEVEL (S.1 – S.4) ………………….4<br>O' LEVEL PROFESSIONAL …………5<br>A' LEVEL (S.5 – S.6) …….…………6<br>UNIVERSITY…………………….…..…7<br>OTHER TERTIARY (AFTER S.6) ..….8<br>OTHER (Specify) ________________9	
1407	Who is the MAIN household income earner?	Father ……………………….…1<br>Mother………………………….2<br>Relatives……………………….3<br>Children (6 – 17) years ……..4	

		Grand or Elderly Parents…….5	
1408	What is the MAIN SOURCE of household income? (emphasis is main source only)	Formal Employment / Wage ………………1<br>Commercial Farming ………………………2<br>Formal Business …………….…………...3<br>Petty Business<br>Peasantry/hiring out labour on other farms / gardens ……………………………………..4<br>Informal Employment ……………………...5<br>Causal Labourer …………………………...6<br>Remittances ……………………….…….7<br>None ………………………………..……….8	
1409	Does this household have access to land?	Owns and able to access land ……………..1<br>Owns but not able to access land …………2<br>Does not own, but able to access land……3<br>Does not own, not able to access land……4	
1410	In the last 12 months (MENTION THE MONTH), did the household experience any adverse event that led to an economic loss? (e.g. job loss, death in household, migration, loss of property, etc.)	YES . . . .…. . 1<br>NO . . . .….. 2	
	CPA 2: FOOD SECURITY AND NUTRITION		
1411	Over the past month (MENTION THE MONTH), what has been the MAIN source of food consumed by your household?	Home grown……………………………..…..1<br>Bought from the market…………………..…..2<br>Given in return for work…………………….3<br>Donated……………………………..……….4	
1412	Over the past month, did anyone in the household ever go without food for a whole day because there wasn't enough?	Never…………………………………......1<br>Yes, 1 – 4 times a month……………..2<br>Yes, more than 5 times a month……..3	
	CPA 3: HEALTH, WATER, SANITATION AND SHELTER		
1413	What is the distance (in Km) to the health care facility your household often uses?	Less than 2 km or 1 mile……………..1<br>2 – 5 km or 1 – 2 miles……………….2<br>More than 5 Km or miles……………..3	
1413	Does the household head or caregiver have any form of disability that's severe enough to affect their daily activities? (e.g., physical, speech, visual, hearing, or mental handicap?)	YES . . . . 1<br>NO . . . . …. 2	
1415	What is the main source of water for members of your household?	Private Connection……………………1<br>Public taps, Bore hole, Rainwater, Protected spring/well, Gravity flow scheme …2<br>River, Stream, Lake, Pond, Unprotected well / spring………………………………..3	
1416	How long does it take to collect water for domestic use from the main source? (Time in minutes) Consider time TO & FROM the source of water, INCLUDING waiting time.	15 minutes or less ……………………1<br>16 – 30 minutes ……………………….2<br>More than 30 minutes ………………...3	
1417	What is the MAIN type of dwelling?	Permanent (Sand brick cement)……….1<br>Semi-permanent (mud, iron sheet)…….2<br>Temporary (mud, grass and wattle)…...3	
1418	What is the type of a latrine/toilet facility used by members of your household?	Functional flush toilet, VIP……………..1<br>Pit Latrine / Public toilet……………….2<br>Bush……………………………………...3<br>Other (Specify) _________________ 4	
	CPA 5: PSYCHOSOCIAL SUPPORT AND BASIC CARE		
1419	In the last year, how often have you felt so troubled that you felt you needed to consult a spiritual, faith or traditional healer, counsellor or health worker?	Never………………………………1<br>Sometimes…………………………2<br>Most of the time…………………..….3	
	CPA 6: CHILD PROTECTION AND LEGAL SUPPORT		
1420	What would you do if any of you experienced or became a victim of any form of child abuse or violence?	Report to LC/Police/Probation, CDO, Human rights office……………………….……1<br>Talk to neighbour / family only…………2	

		Nothing…………………………………..3	
1421	In the past 12 months (STATE MONTH), have you or another adult in the household used the following method of discipline with any child in your household? (Please select all the methods that apply)	A. Punched, kicked or hit a child<br>YES . . . . 1<br>NO . . . . 2<br><br>B. Withheld a meal to punish a child<br>YES . . . . 1<br>NO . . . . 2<br>C. Using abusive words/language towards the child<br>YES . . . . 1<br>NO 2	

INTERVIEW ENDED AT: ____AM/PM:

THANK YOU SO MUCH FOR YOUR TIME

THE END
