# Supplementary material for: Sexual-risk behaviours and HIV and syphilis prevalence among in- and out-of-school adolescent girls and young women in Uganda: A cross-sectional study
Source: PLoS One. 2021 Sep 10;16(9):e0257321. doi: 10.1371/journal.pone.0257321 (PMC8432796; doi:10.1371/journal.pone.0257321)
Supplement: Attachment — Submitted filename: Reviewer comments.docx [file pone.0257321.s003.docx]

PLOS ONE MANUSCRIPT

**Title:** Sexual-risk behaviours and HIV and syphilis prevalence among in- and out-of-school adolescent girls and young women in Uganda: a cross-sectional study

**Summary**

This is a cross-sectional study in which the authors sought to compare and link sexual risk behaviours in school going and out-of-school adolescent girls and young women in Uganda with HIV and syphilis biomarkers. The study strengths lie in the large sample size and the wide age group range that caters for different subgroups. The study is important as it seeks to encourage target specific interventions against sexual risk behaviours. The authors reported differences in sexual risk behaviours and HIV and syphilis between the different study groups but did not include enough statistical data to indicate if the differences were significant. I recommend the following revisions and clarifications:

**Specific recommendations**

1. Abstract - Include introduction statement/background information before getting to the study aim.
2. Some Risk ratios in abstract results do not appear in the main results section
3. Questionnaire validation - How many and how were participants selected for field pilot studies. Please indicate
4. Line 245 indicates that editing of questionnaires was done in the field. Wasn’t the questionnaire finalized before beginning of field work and how were differences in the questionnaires between field teams handled?
5. Line 309 – For the statement, “rejecting the two most common misconceptions about HIV transmission or prevention”. What were these misconceptions? Please indicate
6. Line 457 – HIV and wealth tertile, Please indicate if this difference was significant
7. Table 4 heading – Please revise heading by additionally including link to sexual risk behaviours
8. Table 4 – Please indicate if reported differences were significant
9. Discussion – Line 477 – 487 and 580 - 584. It’s important for these findings and conclusions to be supported by statistical analysis indicating if differences between the groups were significant. This is especially so since the major aim of the study was to link sexual risk behaviours with biomarkers in terms of HIV and syphilis prevalence

**Minor comments**

Remove duplicated words, “to reduce” in the abstract conclusion - line 69
